# Chronological, geographical, and seasonal trends of human cases of avian influenza A (H5N1) in Vietnam, 2003–2014: a spatial analysis

**DOI:** 10.1186/s12879-016-1391-8

**Published:** 2016-02-04

**Authors:** Toshie Manabe, Kazue Yamaoka, Toshiro Tango, Nguyen Gia Binh, Dao Xuan Co, Nguyen Dang Tuan, Shinyu Izumi, Jin Takasaki, Ngo Quy Chau, Koichiro Kudo

**Affiliations:** 1 Teikyo University, Graduate School of Public Health, 2-11-1 Kaga, Itabashi-ku, Tokyo, 173-8605 Japan; 2 Department of Hygiene and Public Health, Teikyo University School of Medicine, Tokyo, Japan; 3 Waseda University, 1-21-1 Nishi-Waseda, Shinjuku-ku, Tokyo, Japan; 4 University of Tsukuba, Graduate School of Comprehensive Human Sciences, Ibaraki, Japan; 5 Center for Medical Statistics, Tokyo, Japan; 6 Bach Mai Hospital, Intensive Care Unit, 78 Giai Phong, Dong Da, Hanoi, Vietnam; 7 National Center for Global Health and Medicine, Division of Pulmonary Medicine, Tokyo, Japan; 8 Department of Pulmonary Medicine, Bach Mai Hospital, 78 Giai Phong, Dong Da, Hanoi, Vietnam; 9 Koto Hospital, 6-8-5 Ojima, Koto-ku, Tokyo, Japan

**Keywords:** Avian influenza, H5N1, Geographical trend, Spatial analysis, Disease clustering, Seasonal variation

## Abstract

**Background:**

Human cases of highly pathogenic avian influenza A (H5N1) virus infection continue to occur in Southeast Asia. The objective of this study was to identify when and where human H5N1 cases have occurred in Vietnam and how the situation has changed from the beginning of the H5N1 outbreaks in 2003 through 2014, to assist with implementing methods of targeted disease management.

**Methods:**

We assessed the disease clustering and seasonal variation of human H5N1 cases in Vietnam to evaluate the geographical and monthly timing trends. The clustering of H5N1 cases and associated mortality were examined over three time periods: the outbreak period (2003–2005), the post-outbreak (2006–2009), and the recent period (2010–2014) using the flexibly shaped space-time scan statistic. The most likely cases to co-cluster and the elevated risks for incidence and mortality were assessed via calculation of the relative risk (RR). The H5N1 case seasonal variation was analysed as the cyclic trend in incidence data using Roger’s statistical test.

**Results:**

Between 2003 and 2005, H5N1 cases (RR: 2.15, *p* = 0.001) and mortality (RR: 2.49, *p* = 0.021) were significantly clustered in northern Vietnam. After 2010, H5N1 cases tended to occur on the border with Cambodia in the south, while H5N1 mortality clustered significantly in the Mekong delta area (RR: 6.62, *p* = 0.002). A significant seasonal variation was observed (*p* < 0.001), with a higher incidence of morbidity in December through April.

**Conclusions:**

These findings indicate that clinical preparedness for H5N1 in Vietnam needs to be strengthened in southern Vietnam in December–April.

**Electronic supplementary material:**

The online version of this article (doi:10.1186/s12879-016-1391-8) contains supplementary material, which is available to authorized users.

## Background

In Southeast Asia, including in Vietnam, human cases of highly pathogenic avian influenza A (H5N1) virus infection continue to occur and have been associated with high mortality rates [1]. The current increased number of cases in Egypt [1, 2] should encourage clinicians and policy makers to enhance the clinical and public health preparation in each country and region for H5N1 infection as well as for a potential future influenza pandemic.

Human cases of H5N1 infection often have a rapid progression and cause high mortality [3–6]. Previous studies indicate that early diagnosis and administration of antiviral agents relate to patient survival [7–11]. In many developing countries, the diagnosis using real-time reverse-transcriptase polymerase chain reaction (RT-PCR), one of the most sensitive methods for detecting influenza virus, is only possible in centralised locations with well-trained staff. Additionally, limited medical resources, including antiviral drugs, often delay the initiation of treatment [12]. Furthermore, patients frequently need to travel hundreds of kilometres to receive the appropriate medical services [13]. Education for residents in high-risk areas is a good way to provide the appropriate knowledge for preventing H5N1 infection [14], but it is difficult to provide these programs for all residents. Without an in-depth analysis, human cases of H5N1 appear to occur sporadically. However, we hypothesized if a spatial and temporal pattern of human H5N1 cases can be discovered, more tailored clinical preparedness plans will be possible, including preventive measures, the effective allocation of medical and human resources, and the provision of education programs.

Direct or indirect contact with sick or dead domesticated poultry is a potential cause of H5N1 infection in humans [15]. As a result, previous studies have examined the circulation and movement of poultry flocks across nations and performed spatial and temporal analyses of H5N1 outbreaks among birds in Vietnam [15–17]. One study examining eight countries in Southeast Asia reaffirmed that human H5N1 cases and H5N1 outbreaks among poultry often co-occur seasonally [18]. Understanding the spatial and temporal patterns of H5N1 outbreaks among poultry is crucial for understanding the spatial and temporal patterns of human H5N1 cases. However, a detailed evaluation using spatial and temporal analyses of the timing and location of human H5N1 cases in Vietnam has not been reported.

The aims of the present study were to use space-time analyses to evaluate when and where human H5N1 cases have occurred in Vietnam and how the situation has changed from the beginning of the H5N1 outbreaks in 2003 to the present. The results may contribute toward more directed and targeted interventions for preventing and controlling the spread of H5N1 human infections.

## Methods

A space-time analysis was conducted to evaluate the disease clustering and mortality of human cases of H5N1 infection as well as the seasonal variation of these cases in Vietnam. The seasonal variation was defined as a cyclic trend of incidence on a monthly basis [19]. The incidence and mortality data were collected from case reports of H5N1 human infections by the World Health Organization (WHO) and the WHO Western Pacific Region between December 2003 and December 2014 [20, 21].

Data on the location (province) of occurrence, dates of disease onset and hospital admission, and outcome (death or survival) of each human H5N1 case were collected.

Because a reporting system with detailed information on H5N1 patients had not yet been established during the 2003–2005 outbreak period, cases from this time were only included in our study if they were confirmed by RT-PCR. These cases were used in the disease clustering analysis only if the name of the province where the case occurred and the patient outcome were both reported, and they were used for the seasonal variation analysis only if the date of disease onset, hospitalization, or death were reported clearly. In contrast, all of the cases from 2006 to 2014 were included in the present study. Thus, the number of included cases from the 2003–2005 outbreak period used in analysing the seasonal variation and the disease clustering was 74 and 68 cases (36 fatal), respectively. According to the WHO, however, the number of reported cases in Vietnam during this period was 93 (42 fatal). After the reporting system was established in late 2005, detailed information on all H5N1 cases in Vietnam were provided from the WHO. Thus, from 2003 to 2014, a total of 108 cases were analysed for seasonal variation and 98 cases (60 fatal) for disease clustering. A sub-analysis to determine the sensitivity of our study was conducted with the data from 2006 through 2014 because the data from these years was complete for all reported H5N1 cases.

The population of each Vietnamese province was obtained from a 2012 report by the General Statistics Office of Vietnam [22] and used to analyse a standardised morality ratio (SMR) and a standardised incidence ratio (SIR) of disease clustering [23]. The SMR and SIR for each province in Vietnam were then plotted on a map. Geographical information, including the latitude and longitude of each province and its neighbouring provinces, was also collected for our disease mapping. The collected cases were divided into three time periods: the outbreak period (2003–2005), the post-outbreak period (2006–2009), and the recent period (2010–2014). These time periods were chosen to evaluate possible changes over time as well as to evaluate the effects of a strategic disease control project being conducted in northern Vietnam that our group began in 2010 [9, 14, 24, 25].

### Statistical analyses

Disease clustering was detected using the flexibly shaped space-time scan statistic (FleXScan) [23, 26–29]. The FlexScan analysis was implemented with the restricted likelihood ratio proposed by Tango [23, 29]. It was shown that as the relative risk (RR) of the cluster becomes large via a Monte Carlo simulation, this method is able to detect clusters of any shape reasonably well [23, 26]. We selected a flexible spatial scan as our scanning method. A Poisson model was used for spatial analysis scanning for clustering. The *p*-values were calculated using the original log likelihood ratios. To characterise a detected spatial cluster, we estimated the RR, the SIR, and the SMR. The SIR and SMR are the observed number of cases divided by the expected frequency of incidence and mortality, respectively, within the clusters. For the circulating spatial scan statistic, the maximum length of the geographical window was set to 20 of the nearest neighbours [23, 26]. The number of replications for the Monte Carlo procedure was set to 999. Seasonal variations in the monthly incidence of human H5N1 infection were evaluated using the number of cases according to the month of disease onset or hospital admission. To evaluate if there was a seasonal variation in the incidence data, we used the cyclic trend significance test proposed by Roger [30]. In this method, the null hypothesis is that the incidences are equally likely to be allocated in each of the months.

Data analyses were conducted using FleXScan version 3.1 [28] and SAS version 9.3 (SAS Institute Inc., Cary, North Carolina). The *p*-values were two-tailed, and *p* < 0.05 was considered significant.

## Results

### Human cases of avian influenza H5N1 infection in Vietnam

**Fig. 1:**
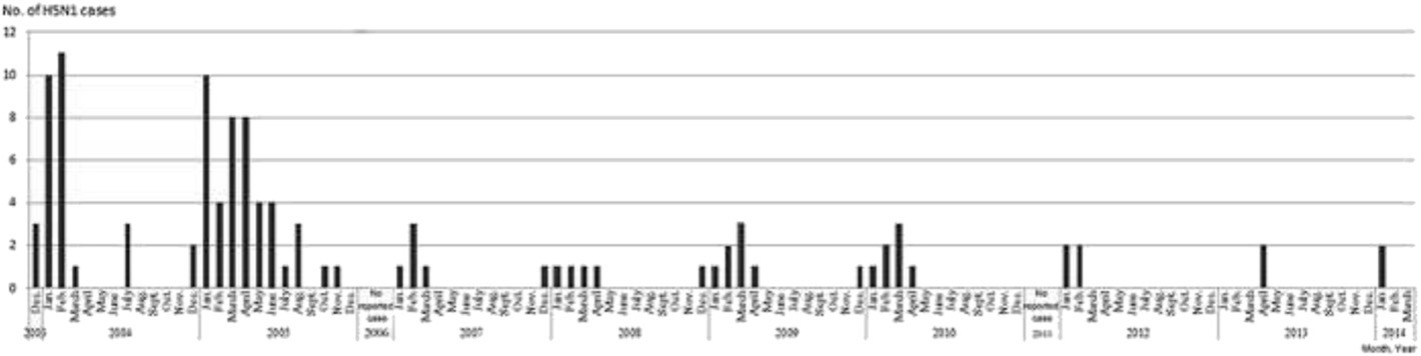
Observed human cases of H5N1 infection from December 2003 to December 2014

Between December 2003 and December 2014, a total of 127 confirmed human H5N1 cases were reported to the WHO from Vietnam, 64 of which were fatal [1]. The highest numbers of cases were reported between 2003 and 2005 (Fig. 1). Although there were no cases reported in 2006 or 2011, some cases were reported in 2014.

### Seasonal trends of human H5N1 cases in Vietnam

**Fig. 2:**
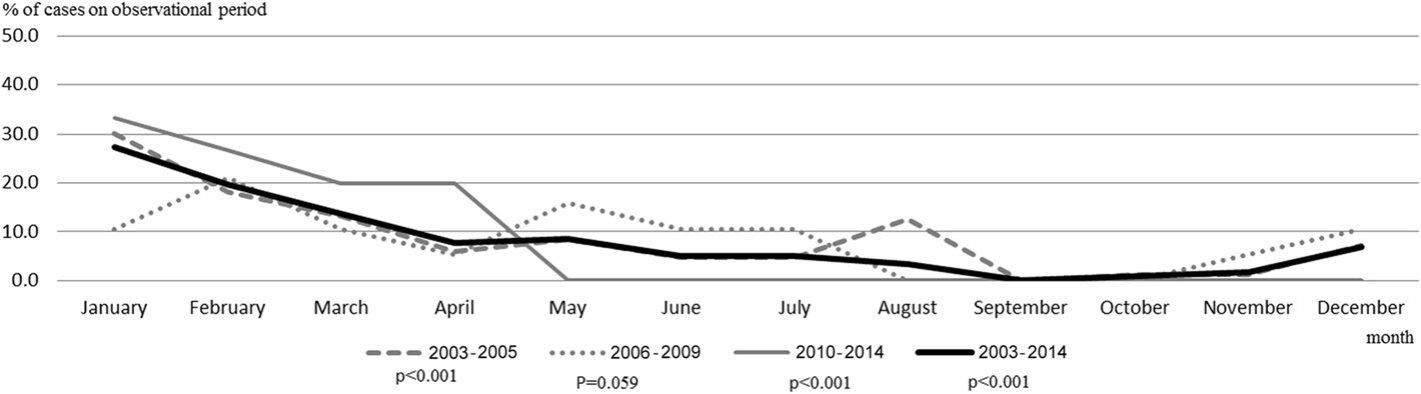
Seasonal variation during the entire observational period (2003–2014) was tested by Roger’s statistic (*p* < 0.001). The *p* values for each period were *p* < 0.001 in the outbreak period (2003–2005), *p* = 0.059 in the post-outbreak period (2006–2009), and *p* < 0.001 in the recent period (2010–2014)

To investigate the seasonal variation in human H5N1 cases, we evaluated the trends in the monthly timing of these cases. A significant seasonal variation was observed from the beginning of the H5N1 outbreak in 2003 to the end of 2014, (*p* < 0.001), and from 2006 to the end of 2014 (*p* < 0.001) (Fig. 2). Similarly, the seasonal variations within the outbreak period (2003–2005) (*p* < 0.001) and the recent period (2010–2014) (*p* < 0.001) were significant, and although there was a trend toward seasonal variation within the post-outbreak period (2007–2009), this failed to reach statistical significance (*p* = 0.059). Compared with the other months, H5N1 cases occurred at a higher incidence between December and April and a lower incidence between August and November.

### Geographical disease clustering of human H5N1 infection incidence and mortality in Vietnam

**Fig. 3:**
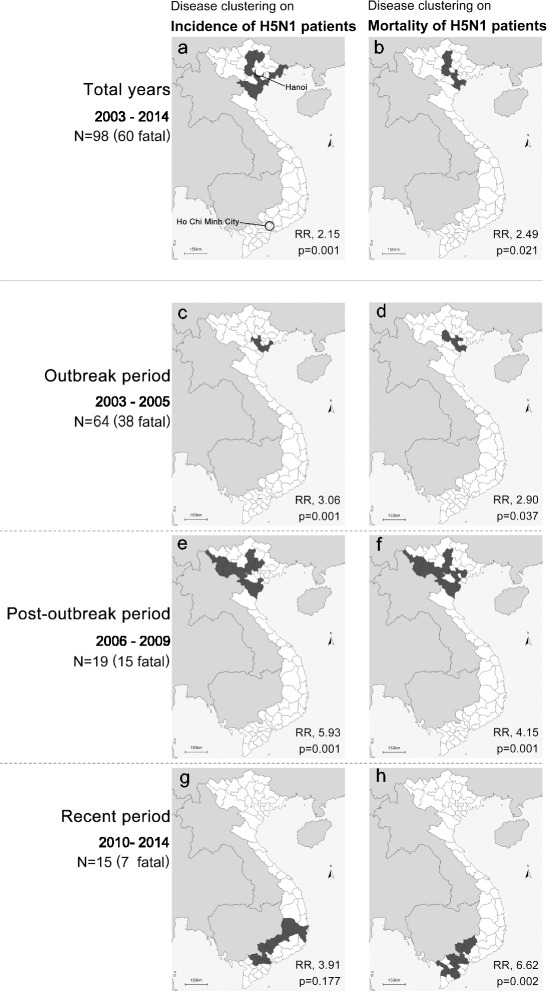
Clustering of the incidence and mortality of human H5N1 in Vietnam from 2003 to 2014. Clustering of H5N1 cases (left panels) and mortality (right panels) in 2003–2014 **a**, **b**, 2003–2005 **c**, **d**, 2006–2009 **e**, **f**, and 2010–2014 **g**, **h**. Black areas represent the clusters of human H5N1 incidence (left panels) and mortality (right panels) due to H5N1 infection during each time period

Disease and mortality clustering of H5N1 cases was shown in Fig. 3. From 2003 to 2014, the incidence (Fig. 3a) and mortality (Fig. 3b) of H5N1 cases were significantly clustered in the central area of northern Vietnam and in the rural mountainous and coastal areas of northern Vietnam, respectively. During the H5N1 outbreak period (2003–2005), a cluster of H5N1 cases was detected in the central area of northern Vietnam (Fig. 3c) and the H5N1 case mortality was clustered in the central and coastal areas (Fig. 3d). In the post-outbreak period, the incidence of H5N1 cases (Fig. 3e) and H5N1 mortality (Fig. 3f) spread to the agricultural areas in northern Vietnam other than the central areas of Hanoi. From 2010 to 2014, there was no significant clustering of H5N1 cases, only a trend toward cases being predominantly located in the areas that border Cambodia in southern Vietnam (Fig. 3g). During this period, a significant cluster of H5N1 mortality was detected near the border of Cambodia and the Mekong delta in southern Vietnam (Fig. 3h).

We also performed a subgroup analysis using data from 2006 through 2014 because the data from the outbreak period (2003–2005) did not include all reported cases, as described in the Methods section. The H5N1 cases (RR: 4.15, *p* = 0.001) and H5N1 mortality (RR: 4.55, *p* = 0.044) from this period were significantly clustered in northern Vietnam (Additional file 1).

## Discussion

The present study revealed that H5N1 mortality in Vietnam was significantly clustered in the northern part of Vietnam from 2003 through 2014, but during the recent period, H5N1 mortality has clustered in the southern part of Vietnam. We also observed a significant seasonal variation in the human H5N1 cases, with a higher disease incidence in December through April.

Large outbreaks of human H5N1 cases occurred toward the end of 2003, simultaneous with large H5N1 outbreaks among poultry throughout Asia [6, 31, 32]. Since then, a history of contact with infected poultry has been reported for many of the human H5N1 cases and is believed to be one of the risk factors of human H5N1 infection [7, 15, 33–35]. Therefore, the seasonal variation in human H5N1 cases might correspond to the time when migratory birds typically infect domesticated poultry with H5N1 [36]. The present study has shown that human H5N1 cases in Vietnam are most common from December to April (Fig. 2). This timing overlaps with that of H5N1 infections in poultry, which typically occur between January and March [18]. These results indicate that the clinical preparedness and disease monitoring for H5N1 should be enhanced during these overlap months.

Temperature may also be a factor for H5N1 outbreaks among poultry. A previous report indicated that avian influenza virus replication increases at cooler temperatures [37], and colder weather may enable prolonged viral survival in the secretions and faeces of infected poultry [18, 38]. The temperature and humidity in Vietnam between December and March are lower than those in the other months, especially in northern Vietnam, which has a subtropical climate [39]. Temperature and humidity are known ecological risk factors for seasonal influenza; therefore, it is likely that they similarly influence the occurrence of human H5N1 cases [40–43]. The seasonal variation in the number of human H5N1 cases typically overlaps with the period of seasonal influenza incidence.

Another possible reason for the seasonal variation of H5N1 infection, possibly unique to Vietnam, may be the increases in poultry population density and trafficking due to the Tet holiday festival (the Lunar New Year of Vietnam), which takes place in January or February of each year. During this period, the distribution, trading, and consumption of chickens increase dramatically because chicken is a traditional dish for the celebration of Tet [44]. Thus, the risk of H5N1 human infection is likely to increase dramatically around the Tet holiday [44]. A similar situation has been suggested for the Chinese New Year festival [45]. The results of our study point to the necessity of understanding the relevant cultural factors to achieve clinical preparedness for avian influenza infection. Although patterns on seasonal variations on each period were similar with the above reasons, the number of incidence of H5N1 human infection has been decreasing. It may be caused by no evidence of human-to-human spread [46].

In Vietnam, human H5N1 cases and mortality were significantly clustered in the coastal and mountainous areas of northern Vietnam from 2003 through 2014 (Fig. 3a, b). At the beginning of the human H5N1 outbreaks between 2003 and 2005, H5N1 cases were reported both from the southern and northern parts of Vietnam [19]. However, significant clusters of H5N1 cases and mortality were detected in northern Vietnam during this period (Fig. 3c, d). Since then, although the number of confirmed human H5N1 cases of has been lower, human H5N1 cases have still occurred sporadically and continued to be present through 2014. In 2005, Vietnam conducted nationwide vaccination programs against poultry that were thought to contribute to the absence of widespread outbreaks in poultry [33]. It might affect to occurrence on human H5N1 cases throughout 2006 [17]. After 2007, significant clusters of H5N1 cases and mortality were still observed in northern Vietnam, but they moved to more rural and mountainous areas. After 2010, no significant clusters of H5N1 cases were found, although there was a trend toward cases being located near the border with Cambodia in southern Vietnam. However, a significant cluster of H5N1 mortality was found during the recent period in the Mekong delta areas in the south.

The outbreak of human H5N1 cases started in December 2003, and cooler temperatures in northern Vietnam may have led to more cases in that area than in southern Vietnam, which has a tropical climate. However, the movement of H5N1 mortality clusters over time may also be strongly influenced by reporting systems, medical techniques and resources, and/or information availability, rather than climatic factors. For example, in the beginning of the H5N1 outbreak, H5N1 cases and mortality were primarily located in and around the central city of Hanoi. The reporting system of Hanoi may be more efficient than those in rural areas, and the RT-PCR technique is more common in Hanoi. Additionally, Hanoi’s hospitals have the medical and human resources to provide advanced medicine for treating patients with unknown serious diseases, and patients with serious conditions are better able to access hospitals in the central city.

There were no significant clusters of H5N1 cases from 2010 to 2014 (Fig. 3g), but a significant cluster of H5N1 mortality was detected during this time period in the area bordering Cambodia and the Mekong delta area in southern Vietnam (Fig. 3h). This cluster of mortality led to a high RR (6.62) compared with those of other periods. Since 2011, the numbers of reported human H5N1 cases and mortality in Cambodia have increased dramatically [1]. The movement of humans and trade, including poultry, between Cambodia and Vietnam might affect the occurrence of human H5N1 cases in areas close to the border of Cambodia [40]. This area is also thought to have fewer medical and human resources and less clinical information and support for treating H5N1 patients. The other neighbour countries including Thailand and Lao did not report H5N1 in human cases since 2007, and China has been reporting continuously; however, the number of reported cases was less than 2 in 2010–2014 [1]. The situation of neighbour countries may not affect to those of Vietnam other than Cambodia. Starting in 2010, our group has conducted an H5N1 treatment network program, which includes education for residents and medical providers as well as clinical consultations for clinicians in the local hospitals in northern Vietnam [9, 20–22]. This network may have helped to reduce the mortality from human H5N1 in the north and could be one of the factors that led to the cluster of H5N1 mortality moving from northern to southern Vietnam.

One limitation of the present study is that the collected data are from the WHO’s official reports of human H5N1 cases. It is possible that the number of cases reported to the WHO is underestimated. Additionally, some of these reports lack detailed information. Consistent monitoring activities on a local basis would help with obtaining a more accurate evaluation of H5N1 human incidence and mortality. Another potential limitation of this study could be our use of the 2012 Vietnamese population throughout all the observational years for our SIR and SMR analyses of disease clustering; however, the mean population over these years was similar to the population during 2012 and it has not changed drastically over this time period.

## Conclusion

In conclusion, the results of the present study provide statistically significant evidence of H5N1 disease incidence and mortality clustering. These findings will contribute toward directed and targeted interventions, improving our preparedness and control activities for H5N1 human infections. To reduce the disease severity and number of deaths from H5N1 infection, clinical and public health preparedness for H5N1 in Vietnam should be strengthened from December–April, particularly in the areas bordering Cambodia and the Mekong delta region in southern Vietnam. Continuous and timely disease monitoring are necessary for the detection of H5N1 outbreaks and other novel influenza virus infections throughout Vietnam and may assist in worldwide prevention of influenza infections.

### Ethics approval and consent to participate

The ethical approval was waived by the ethics committee of Teikyo University for this study using open-accessed anonymized information.

## Additional file

Additional file 1:
**Geographical disease clustering of incidence and mortality of human H5N1 infection in Vietnam between 2007 and 2014.** (JPG 113 kb)

## Abbreviations

RT-PCR: real-time reverse-transcription polymerase chain reaction
RR: relative risk
SMR: standardised morality ratio
SIR: standardised incidence ratio
FleXScan: the flexibly shaped space-time scan statistic.
