# Supplementary figures and images for: Chronological, geographical, and seasonal trends of human cases of avian influenza A (H5N1) in Vietnam, 2003–2014: a spatial analysis
Source: BMC Infect Dis. 2016 Feb 4;16:64. doi: 10.1186/s12879-016-1391-8 (PMC4743110; doi:10.1186/s12879-016-1391-8)

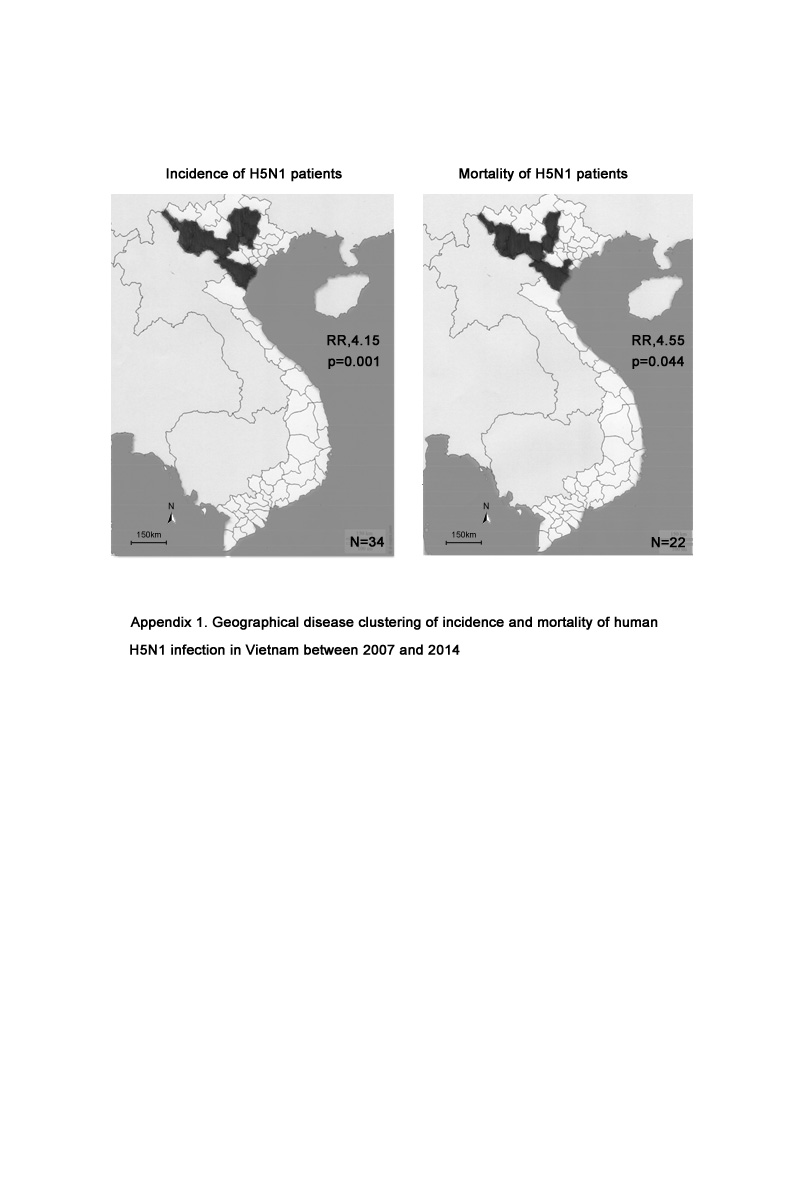

Supplement: Additional file 1: — Geographical disease clustering of incidence and mortality of human H5N1 infection in Vietnam between 2007 and 2014. (JPG 113 kb) [file 12879_2016_1391_MOESM1_ESM.jpg]
